# Improving Phenolic Total Content and Monoterpene in *Mentha* x *piperita* by Using Salicylic Acid or Methyl Jasmonate Combined with Rhizobacteria Inoculation

**DOI:** 10.3390/ijms21010050

**Published:** 2019-12-19

**Authors:** Lorena del Rosario Cappellari, Maricel Valeria Santoro, Axel Schmidt, Jonathan Gershenzon, Erika Banchio

**Affiliations:** 1 INBIAS (CONICET-Universidad Nacional de Río Cuarto), Campus Universitario, 5800 Río Cuarto, Argentina; lcappellari@exa.unrc.edu.ar; 2 Department of Biochemistry, Max Planck Institute for Chemical Ecology, Hans-Knöll-Str. 8, 07745 Jena, Germany; msantoro@ice.mpg.de (M.V.S.); aschmidt@ice.mpg.de (A.S.)

**Keywords:** salicylic acid, jasmonic acid, rhizobacteria, plant growth-promoting rhizobacteria (PGPR), mint, total phenolic content, monoterpene, menthol, pulegone

## Abstract

The effects of plant inoculation with plant growth-promoting rhizobacteria (PGPR) and those resulting from the exogenous application of salicylic acid (SA) or methyl jasmonte (MeJA) on total phenolic content (TPC) and monoterpenes in *Mentha* x *piperita* plants were investigated. Although the PGPR inoculation response has been studied for many plant species, the combination of PGPR and exogenous phytohormones has not been investigated in aromatic plant species. The exogenous application of SA produced an increase in TPC that, in general, was of a similar level when applied alone as when combined with PGPR. This increase in TPC was correlated with an increase in the activity of the enzyme phenylalanine ammonia lyase (PAL). Also, the application of MeJA at different concentrations in combination with inoculation with PGPR produced an increase in TPC, which was more relevant at 4 mM, with a synergism effect being observed. With respect to the main monoterpene concentrations present in peppermint essential oil (EO), it was observed that SA or MeJA application produced a significant increase similar to that of the combination with rhizobacteria. However, when plants were exposed to 2 mM MeJA and inoculated, an important increase was produced in the concentration on menthol, pulegone, linalool, limonene, and menthone concentrations. Rhizobacteria inoculation, the treatment with SA and MeJA, and the combination of both were found to affect the amount of the main monoterpenes present in the EO of *M. piperita*. For this reason, the expressions of genes related to the biosynthesis of monoterpene were evaluated, with this expression being positively affected by MeJA application and PGPR inoculation, but was not modified by SA application. Our results demonstrate that MeJA or SA application combined with inoculation with PGPR constitutes an advantageous management practice for improving the production of secondary metabolites from *M. piperita*.

## 1. Introduction

Modern agriculture does not restrict itself to traditional food, forage, and fiber crops; rather, there is an increasing interest in crops that include species with secondary metabolites. These are present in all plant tissues at variable concentrations. They are low molecular weight compounds that are very important in plant ecology because they are responsible for the processes of adaptation of plants to their environment. Given their diverse biological and physio-chemical properties, secondary metabolites are of great interest as unique sources for pharmaceuticals, food additives, oils, waxes, perfumes, flavoring agents, dyes, and many other commercially important materials [1,2,3].

Peppermint (*Mentha* x *piperita* L.; family Labiatae) is an important, commonly-used flavoring agent world-wide. *M. piperita* plants contain ~3% volatile oils, consisting of >50 different compounds. The major EO components, which make up ~60% of its total oil volume, are limonene, linalool, menthone, menthol, and pulegone. Peppermint leaves are used for teas and flavoring foods and beverages, and its EOs are also used in chewing gum, candy, toothpaste, mouthwash, aromatherapy, pharmaceuticals, antimicrobial agents, and eco-friendly pesticides [4]. Not less important components of peppermint leaves are the phenolic compounds, including caffeic acid, rosmarinic acid, eriocitrin, and luteolin-7-*O*-glucoside [5,6], which represent about 20% of the dry weight. Seventy-five percent of these compounds can be extracted in an infusion [7,8,9,10]. The infusion of peppermint leaves is a common beverage with a refreshing flavor and a particular fragrance.

Many natural compounds extracted from plants have shown biological activities. There is a growing consumption of natural products with potential health benefits, and novel bioactive compounds are continually being discovered. Among others, plants are regarded as major sources of bioactive compounds with anticancer, antioxidant, antimicrobial, and anti-inflammatory effects [11,12]. Phytobioactive compounds commonly play important physiological roles in plants as secondary metabolites, which can be modified quantitatively and qualitatively through environmental changes as well as exposure to biotic and abiotic stress [13].

As knowledge has grown of the biological functionalities of secondary metabolites, so too has the search for new biotechnological alternatives to improve the production of economically important secondary metabolite compounds [2,3,14,15,16] with bioactive properties which can be used in agriculture as herbicides and pesticides, or as medicinal agents. Several strategies have been investigated to enhance the production of secondary metabolites from medicinal plants, including high-yielding cell line screening, media modification, elicitation, precursor feeding, large-scale cultivation systems, plant cell immobilization, hairy root culture, biotransformation, and others [17,18].

Jasmonic acid (JA), methyl jasmonate (MeJA), and salicylic acid (SA) are known as potent elicitors and plant defense hormones, which play significant roles in regulating plant defense responses against biotic and abiotic stress [19]. JA and its related compounds have long been known as transducers of elicitor signals for the production of plant secondary metabolites [20]. Exogenous application of JA signaling compounds stimulates the biosynthesis of secondary metabolites including a wide variety of plant secondary products such as terpenoids, flavonoids, alkaloids, and phenylpropanoids [21,22], while MeJA application has been reported to be both safe and inexpensive [23,24]. The JA signaling pathway is generally regarded as an integral signal for biosynthesis of many plant secondary products. Also, as many elicitors stimulate endogenous JA biosynthesis in plants, the JA signaling pathway is regarded as a transducer or mediator for elicitor signaling [25].

Salicylic acid (SA) is a well-known inducer of plant systematic acquired resistance (SAR) in plant–pathogen interaction, but it is not a universal inducer for the production of plant defensive metabolites. SA quickly accumulates at the infection site during pathogen attack and plant hypersensitive reaction, and it spreads to other parts of the plant to induce a wide range of defense responses, leading to the accumulation of plant secondary metabolites [26,27,28].

It is well known that a group of bacteria colonize the root systems of plants and can modulate plant growth, with beneficial effects on plant growth and crop yield and quality. Such bacteria, collectively termed “plant growth-promoting rhizobacteria” (PGPR), promote plant growth by enhancing the availability of nutrients, inducing metabolic activities by phytohormones and analogs, by shifting the phytohormonal balance, by inducing systemic resistance defense (ISR), or by reducing phytotoxic microbial communities [29,30,31]. Depending on the PGPR species, two or more of these growth-promoting mechanisms may be present [31,32,33,34,35].

Our previous studies showed that PGPR inoculation increased secondary metabolite production in various aromatic plant species [36,37,38]. The same beneficial bacteria inoculated in different plant species increased total EO yield (relative to controls), but not necessarily at the same level [37,38,39]. We observed that the effects of rhizobacteria on these secondary metabolites varied depending on the strain, suggesting that the rhizobacteria are recognized by the host plant in a strain-specific manner.

Thus, the elicitation of secondary metabolites appears to be a promising and innovative alternative. There are combinations of both biotic and abiotic forms of elicitors that stimulate metabolism but, while there are many studies of their potential use in improving secondary metabolism biosynthesis individually, few reports were found with the combination of both. Knowing that exogenous MeJA and SA applications, such as inoculation with PGPR, increase secondary metabolites, we inoculated the plants with PGPR and simultaneously applied phytohormones directly to learn how the combination of treatments affects total phenolic content (TPC) and the main monoterpenes of *Mentha* x *piperita*. New less aggressive biotechnological methods are needed to enhance the production of secondary metabolites from medicinal plant crops based on the use of beneficial microorganisms applied as biofertilizers.

## 2. Results

### 2.1. Total Phenolic Content (TPC)

**Figure 1:**
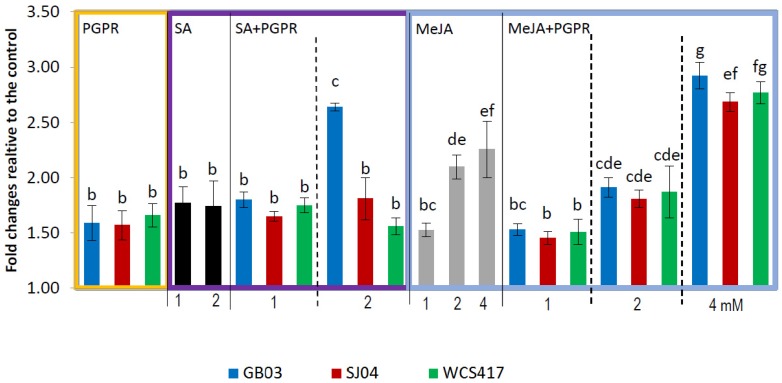
Inoculation with plant growth-promoting rhizobacteria (PGPR) and hormone treatments increase total phenolic compounds content in shoots of *M.* × *piperita* plants. Values are fold changes relative to the control. Different letters above bars for PGPR/*methyl jasmonte (MeJA)* + PGPR/salicylic acid (SA) + PGPR groups indicate significant differences according to the Tukey test (*p* < 0.05). The letter “a” indicates similar to the control. Three different ANOVA analyses were performed (i) PGPR (yellow box), (ii) SA–SA + PGPR (violet box), (iii) MeJA–MeJA + PGPR (blue box). TPC in control plants: for PGPR treatments = 209.84 ± 13.20 µg/g fresh weight, for SA treatments = 254.03 ± 35.26 µg/g fresh weight, for MeJA treatments = 192.43 ± 16.52 µg/g fresh weight. Native values are given in Supplementary Tables S1 and S2.

Phenolic compounds are plant secondary metabolites that can be released under the influence of multiple biotic and abiotic stresses; thus, we sprayed inoculated plants with SA or JA at different concentrations in order to determine the effects of the combination of treatments in TPC. An increased accumulation of total phenols was observed in inoculated mint plants compared to untreated controls (*p* < 0.05) (Figure 1). TPC in leaves of WCS417r inoculated plants showed further increased accumulation of total phenolic compounds (347.98 mg/mg fresh weight); this phenolic accumulation was similar for the three strains evaluated. The TPC in plants sprayed with SA at 1 or 2 mM concentrations showed similar phenolic content to plants only inoculated (~400 mg/mg fw) (Figure 1, Supplementary Table S1). The main effect was observed when plants were treated with 2 mM in combination with GB03 increasing up to 60% compared to individual treatments (2 mM SA or PGPR; *p* < 0.05).The response of plants exposed to MeJA depended on the concentration applied (Figure 1); as the concentration increased, the effect was also greater, almost 2.5 times with 4 mM in relation to control plants. When plants were inoculated and treated with 1 or 2 mM MeJA, the TPC was comparable to that of plants treated only with MeJA regardless of the strain inoculated; when plants were treated with 4 mM+GB03 or WCS417, it increased up to 3 times (613.11–563.22 mg/mg fw; Supplementary Tables S1 and S2) compared to control, and was higher than MeJA alone or inoculated plants.

### 2.2. Phenylalanine Ammonia Lyase Activity (PAL)

**Figure 2:**
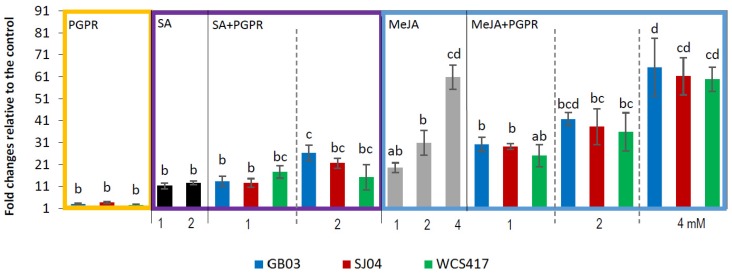
Inoculation with PGPR and hormone treatments modify phenylalanine ammonia lyase (PAL) activity in shoots of *M.* × *piperita* plants. Values are fold changes relative to the control. Different letters above bars for PGPR, MeJA + PGPR, SA + PGPR groups indicate significant differences according to the Tukey test (*p* < 0.05). The letter “a” indicates similar to the control. Three different ANOVA analyses were performed (i) PGPR (yellow box), (ii) SA–SA + PGPR (violet box), (iii) MeJA–MeJA + PGPR (blue box). PAL activity in control plants: for PGPR treatments = 4.62 ± 0.32 µg trans-cinnamic acid min^−1^ mg^−1^protein, for SA treatments = 4.63 ± 0.30 µg trans-cinnamic acid/min × mg protein, for MeJA treatments = 4.60 ± 0.33 µg trans-cinnamic acid/min × mg protein. Native values are given in Supplementary Tables S1 and S2.

Phenylalanine ammonia lyase activity was affected by inoculation and/or exogenous phytohormone application. Significantly higher PAL activity was observed in inoculated plants compared to untreated controls (*p* < 0.05; Figure 2). SA applications led to 10-fold increased activity regardless of the concentration applied (*p* < 0.05; Figure 2). However, when PGPR and 1 mM SA spraying were combined, the rise in PAL activity was similar to the increase seen in plants treated with SA alone (*p* < 0.05), while in plants inoculated with GB03 and treated with 2 mM, the activity was almost double that in 2 mM treated plants. Spraying with MeJA also produced an increase in PAL activity, which was higher at each higher concentration applied, being ~20-, ~30- and ~60-fold higher in plants treated with 2 and 4 mM, respectively, compared to control plants. When PGPR and MeJA spraying were combined, the effect on PAL activity was statistically similar to that of MeJA alone for the different concentrations evaluated, while with 1 mM were slightly higher.

### 2.3. Quantification of Main EO Components

Since we showed in previous studies that PGPR and the exogenous application of SA and MeJA combined with inoculation increased EO content [40], we analyzed the response of the main EO components in plants treated with PGPR in addition to SA or MeJA. GC analysis of the yields of the major EO components ((+)-pulegone, (−)-menthone, (−)-menthol, limonene, and linalool) revealed great differences among plants treated with the phytohormone and control groups. 

**Figure 3:**
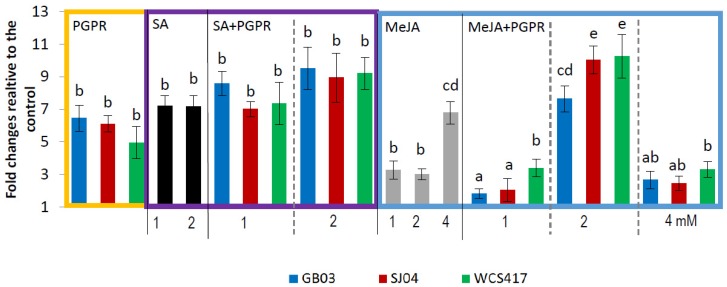
Inoculation with PGPR and hormone treatments increase menthol concentration in shoots of *M.* × *piperita* plants. Values are fold changes relative to the control. Different letters above bars for PGPR, MeJA + PGPR, SA + PGPR groups indicate significant differences according to the Tukey test (*p* < 0.05). The letter “a” indicates similar to the control. Three different ANOVA analyses were performed (i) PGPR (yellow box), (ii) SA–SA + PGPR (violet box), (iii) MeJA–MeJA + PGPR (blue box). Menthol concentrations in control plants: for PGPR treatments = 0.20 ± 0.02 μg/g fw, for SA treatments = 0.19 ± 0.02 μg/g fw, for MeJA treatments = 0.23 ± 0.07 μg/g fw.

**Figure 4:**
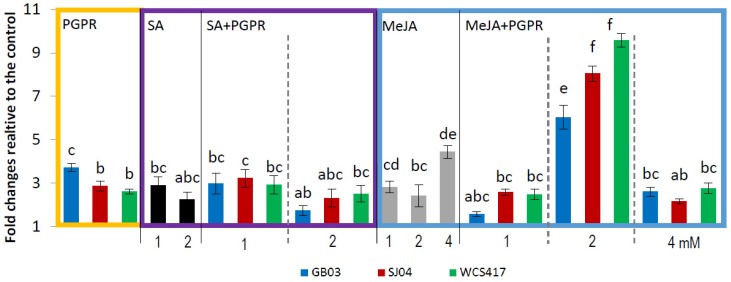
Inoculation with PGPR and hormone treatments increases pulegone concentration in shoots of *M.* × *piperita* plants. Values are fold changes relative to the control. Different letters above bars for PGPR, MeJA + PGPR, SA + PGPR groups indicate significant differences according to the Tukey test (*p* < 0.05). The letter “a” indicates similar to the control. Three different ANOVA analyses were performed (i) PGPR (yellow box), (ii) SA–SA + PGPR (violet box), (iii) MeJA–MeJA + PGPR (blue box). Pulegone concentrations in control plants: for PGPR treatments = 3.74 ± 0.16 μg/g fw, for SA treatments = 4.27 ± 0.27 μg/g fw, for MeJA treatments = 3.99 ± 0.28 μg/g fw.

Plants only inoculated with the different strains produced an increase in all the major monoterpenes evaluated. The total amount of menthol increased (Figure 3) to 1.29 μg/g fresh weight in plants inoculated with GB03 in comparison with 0.20 μg/g fw in the control group (*p* < 0.05). The same trend was observed for pulegone (Figure 4), linalool, limonene, and menthone, where inoculation with GB03 generally produced the greater effect (Table 2).

The external application of SA modified the yield of the main monoterpene compounds of *M. piperita* (Table 1, Figure 3 and Figure 4), but, in general, there was no difference between the application of 1 and 2 mM (*p* > 0.05). The combination of SA with PGPR did not show any significant difference with the application of SA alone for all the compounds evaluated. 

Menthol content in plants sprayed with SA increased 7 times in relation to uninoculated plants (Figure 3), similar values to those in inoculated plants alone (Supplementary Table S1). The same effect was observed for pulegone: SA application increased pulegone approximately three-fold in comparison to the control (Figure 4), similar to the increase in PGPR-inoculated plants (Supplementary Table S1).

Linalool content (Table 1) increased significantly only with 1 mM SA application (*p* < 0.05). 

Limonene content in plants treated with 1 or 2 mM showed the same effect, an increase of 4–5 times compared to controls, a similar effect to inoculated plants (Table 1). 

Menthone yield showed the same trend as limonene. Plants treated with SA showed an increase compared to control plants (*p* < 0.05) (Table 1), but when the treatments were combined, menthone content was similar in plants treated only with SA and in those also inoculated (*p* > 0.05), with the exception of 1mM SA + S, which resulted in a yield increase of 30% with respect to 1 mM SA.

The response of plants treated with MeJA depended on the concentration applied. Plants treated with 1 or 2 mM MeJA increased menthol and pulegone content approximately three-fold in relation to the control, with the greater effect observed in plants treated with 4 mM MeJA (Figure 3 and Figure 4), in which the amounts of menthol and pulegone increased 7 and 5 times, respectively (Figure 3, Supplementary Table S2). The effect of spraying with MeJA and inoculation increased menthol concentration only when 2 mM was applied, reaching 10-fold compared to control plants and 3 times compared to 2 mM-sprayed mint plants, while the combination of 1 or 4 mM and inoculation showed no yield effect compared to sprayed plants alone. A similar effect was observed for pulegone content (Figure 4), where the combination of inoculation with any of the strains evaluated and the application of 1 and 4 mM MeJA showed no significant difference with 1 and 4 mM sprayed plants (*p* > 0.05), while the combination with 2 mM increased pulegone content approximately 6-, 8- and 10-fold in relation to control plants in the inoculated strains, GB03, SJ04 and WCS417, respectively, with higher yields than those obtained with individual treatments (Figure 4; Supplementary Table S2).

Linalool, limonene and menthone content in plants treated with 1 and 2 mM MeJA was not modified (*p* > 0.05) in comparison to control plants, while 4 mM significantly increased the amount of linalool, limonene and menthone, 6-, 3-, and 4-fold, respectively (Table 2). When the inoculation was combined with MeJA, significant differences were observed only at 2 mM, increasing the content of linalool, limonene, and menthone approximately 5-fold in relation to 2 mM-treated plants (Table 2).

### 2.4. PGPR Inoculation and External Phytohormones Application Induces Terpenoid Gene Expression in M. piperita

Both rhizobacteria inoculation and SA and MeJA treatment were found to affect the amount of the main monoterpenes present in the EO of *M. piperita.* We, therefore, evaluated qPCR gene expression of two enzymes involved in the biosynthetic pathway leading to the bioactive monoterpenes. Previous studies have established the biochemical pathway in mints that leads to the production of the most important monoterpenes [41]. We considered the early gene, limonene synthase (*Ls)*, involved in the formation of limonene, which is responsible for the first dedicated step of monoterpene biosynthesis in mint species. Limonene synthase catalyzes the cyclization of geranyl diphosphate, the universal C_10_ precursor of the monoterpenes, to (−)-4-*S*-limonene [41] and one of the end genes, *Pr*, coding for the enzyme pulegone reductase (*Pr*), which produced menthone and isomenthone at an average ratio of 2.5:1 [42].

**Figure 5:**
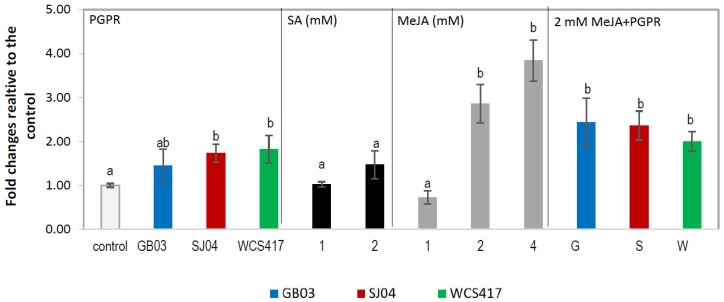
Limonene synthase gene expression of *M.* x *piperita* plants inoculated with PGPR and hormone treatments with respect to control leaves. Bars indicate the standard error over the mean of at least three biological replicates. Means followed by the same letter in a given column are not significantly different according to the Tukey test (*p* < 0.05).

Considering that not all the treatments in the present study produced an increase in the main EO compounds, we determined the gene expression of the most important treatments. *Ls,* coding for the enzyme that catalyzes the conversion of geranyl diphosphate to limonene, one of the simplest of all terpenoid cyclization reactions, was upregulated almost 2-fold in plants inoculated with SJ04 and WCS417(*p* < 0.05). External application of 1 and 2 mM SA had no effect on the expression of *Ls* (*p* > 0.05; Figure 5). In contrast, the application of 2 and 4 mM MeJA upregulated *Ls* expression 3- and 4-fold, respectively (*p* < 0.05). When plants were inoculated and sprayed with 2 mM MeJA, they showed the same effect as with the application of 2 mM alone (Figure 5).

**Figure 6:**
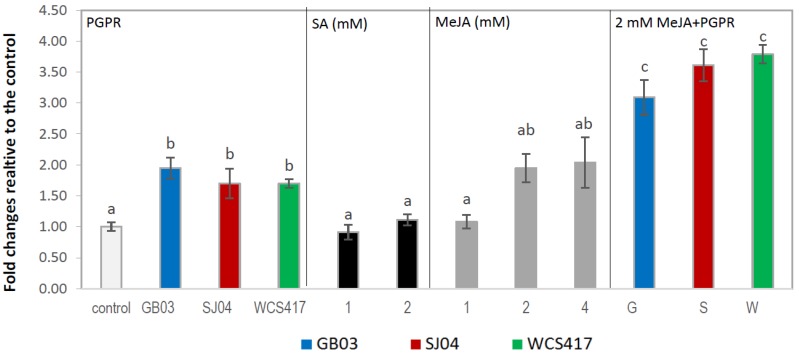
Inoculation with PGPR and hormone treatments increases pulegone reductase expression in shoots of *M.* x *piperita* plants. Values are fold changes relative to the control. Means followed by the same letter in a given column are not significantly different according to the Tukey test (*p* < 0.05). The letter “a” indicates similar to the control.

*Pr*, which codes for pulegone reductase, the enzyme responsible for NADPH-dependent reduction of the conjugated double bond of terpenone to yield (−)-menthone and lesser amounts of (+)-isomenthone [42], was upregulated almost 2-fold by PGPR inoculation (*p* < 0.05; Figure 6). SA did not affect *Pr* expression (*p* > 0.05; Figure 6). MeJA external application produced the same effect as in *Ls* gene expression, which upregulated *Pr* by over 2-fold when it was applied at 2 and 4 mM, but these two values were not statistically different (*p* > 0.05). Moreover, this effect increased (to approximately 3.5-fold) when plants were sprayed with 2 mM MeJA and inoculated. 

### 2.5. Principal Component Analysis

**Figure 7:**
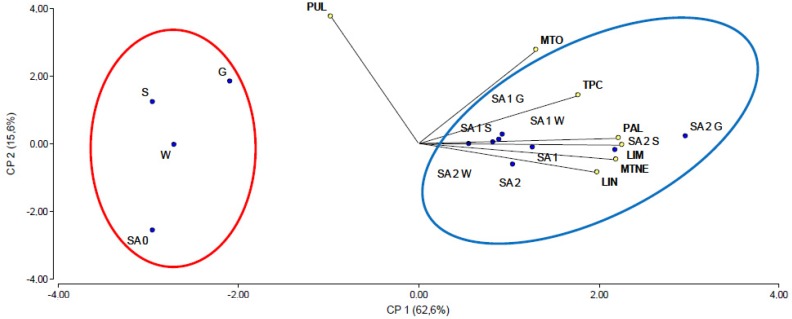
Principal component analysis illustrating relationships among PGR inoculation and SA external application on *M. x piperita* for TPC, PAL and major essential oil components: LIM (limonene content), LIN (linalool content), MTNE (menthone content), PUL (pulegone content), MTO (menthol content).

The PCA was performed to correlate the effects of inoculation with the three PGPR strains and spraying with SA or MeJA on the production of the main monoterpenes, TPC and PAL activity. This type of analysis provides a graph that facilitates the visualization and interpretation of the data set and the variables. Figure 7 shows the PCA correlating the effects of inoculation with the three PGPR strains combined with exogenous SA application. The variation in the data (78.2%) was explained by the first two principal components and gave a cophenetic correlation coefficient of 0.970. This plot shows that the strains, whichever was combined with 1 or 2 mM SA, were located in proximity to all the variables evaluated (blue circle): limonene content (LIM), linalool content (LIN), menthone (MTNE), pulegone (PUL), menthol content (MTO), TPC, and PAL. The control treatments SA and the strains alone are far from the variable evaluated (red circle), showing a low effect in relation to the other treatments. We observed a strong positive correlation (acute angle) between all monoterpene content and TPC content with the exception of PUL content (Figure 7).

**Figure 8:**
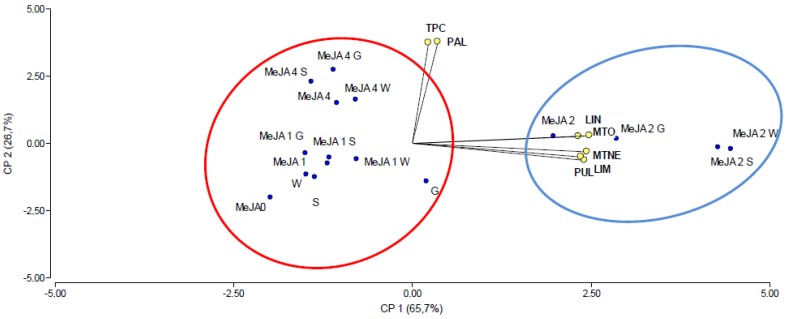
Principal component analysis illustrating relationships among strain inoculation and MeJA external application on *M. x piperita* for TPC, PAL, and major essential oil components: LIM (limonene content), LIN (linalool content), MTNE (menthone content), PUL (pulegone content), MTO (menthol content).

In relation to the PCA that correlates effects of inoculation with MeJA treatment, a plot defined by the first two principal components was sufficient for our purpose because it explained most (92.4%) of the variation in the data and gave a cophenetic correlation coefficient of 0.994 (Figure 8). In the two-dimensional coordinate system based on the first two principal components, it was possible to differentiate the strains combined with different MeJA concentrations. The inoculation and spraying with 1 and 4 mM MeJA were all together, far from the variable evaluated (red circle), while the three strains combined with 2 mM MeJA (blue circle) were located in proximity to the variables LIM, LIN, MTNE, PUL, and MTO. No one treatment was closer to TPC and PAL. In regard to associations among variables, there was a strong positive correlation (acute angle in Figure 8) between the limonene, menthone, menthol and pulegone content, as expected. Surprisingly, no associations (i.e., right angles in Figure 8) were observed between TPC and the major essential oil components.

## 3. Discussion

Plants have been called chemical factories as they have the ability to fabricate important phytochemicals. Changes in secondary metabolites and the enhanced growth of host plants in interaction with different beneficial microbes have recently been studied [30,35]. 

*M. piperita* leaves contain high levels of polyphenolic compounds [5,6]. In the present study, we confirm the strong effect of rhizobacteria inoculation on TPC observed previously in *M. piperita* leaves [43]. The exogenous application of SA produces an approximately 1.5-fold increase in TPC compared to controls, whatever the concentration (1 or 2 mM) applied. In plants inoculated with GB03 and then sprayed with 2 mM SA, TPC increased to a greater extent than with the application of the phytohormone alone (60% higher). These results suggest that the combination produces a synergistic effect on the biosynthesis of phenolic compounds. A similar result was observed for MeJA treatments, where 2 and 4 mM produced an increase of TPC greater than in inoculated plants. Further, when plants were inoculated with GB03 or SJ04 and sprayed with 4 mM, the TPC increased 30% in relation to the individual treatment (MeJA or PGPR), suggesting also a synergism between 4 mM and PGPR inoculation, as observed for 2 mM SA. 

The observed results match those of a previous study in which exogenous applications of JA and SA significantly induced the accumulation of phenolic compounds in a wide variety of plant species [44]. *M. piperita* suspension cultures exposed to JA and MeJA showed increased accumulation of rosmarinic acid (one of the main TPC of peppermint) [45]. The increase in TPC was also observed in suspension cultures supplemented with JA or SA of *Panax ginseng* root [46], of *Thevetia peruviana* [47], *Cucumis melo* [48], in plants of *Romaine lettuce* [49], in buckwheat [50], radish sprouts [51] and *Agastache rugosa* treated only with JA [52]. Similarly, Figueroa Perez et al. [9] reported an increase in TPC of 65%, 35%, and 31% in peppermint treated with SA at 0.05, 0.10, and 0.50 mM, respectively. Similar results were reported in other aromatic plants, such as *Thymus vulgaris* treated with 1 and 2 mM SA [53], *Rosmarinus officinalis* [54], and *Achillea millefolium* [55].

The induction of phenolic accumulation by JA and SA is related with the stimulation of the phenylpropanoid pathway, increasing PAL activity [19,47] in agreement with our observation that PAL activity increases as the concentration of MeJA treatments increases and, for SA treatments, PAL activity increases whatever the concentration applied. Particularly with the combination of 2 mM SA and GB03, the increase of PAL activity was greater than the individual treatments, as observed in TPC content. However, with 4 mM MeJA and inoculation with GB03 or WCS417, PAL activity was similar to that observed for 4 mM treated plants. No synergism in the combination of treatments (4 mM + bacteria) was observed as had been seen in TPC production. 

Kim et al. [49] observed an increase in transcript levels of phenylpropanoid biosynthetic genes after treatment with methyl jasmonate in cell cultures of *Agastache rugosa*. Liu et al. [56] found that 10 µmol JA treatment on pea leaves (*Pisum sativum*) led to a significant increase in the activities of plasma membrane NADPH oxidase and PAL. Jasmonate elicitation was also found to increase the production of phenylpropanoids and naphtodianthrones in *Hypericum perforatum* cell suspensions [57].

Rhizobacteria inoculation was also found to increase TPC and PAL activity in peppermint [43], as was also reported in *Piper betle* inoculated with *Serratia marcescens* and *Tagetes minuta* inoculated with WCS417r and *Azospirillum brasilense* Sp7 [38,58]. The inoculation of chickpea seeds with *P. fluorescens* and *P. aeruginosa*, singly or in combination, induced the synthesis of specific phenolic acids (gallic, ferulic, and chlorogenic) and increased total phenolic content at various stages of plant growth [59]. Salla et al. [60] showed that the inoculation of eucalyptus with *Streptomyces* increased total phenolic content in leaves. In addition, Panka et al. [61] reported that the presence of the endophyte fungus, *Neotyphodium lolii,* increased the content of phenolic compounds in the aerial part of three different genotypes of the perennial grass, *Lolium perenne.* Increased PAL activity was recorded in *P. fluorescens*-pretreated tomato plants challenged with pathogen compared to untreated control [62] and other plant species [63].

Regarding the concentrations of the main monterpene compounds, EO levels and composition in plants play several key roles in plant–environment interactions and plant–plant communication. Terpenoids are crucial components in plant defensive responses to abiotic and biotic stresses [64,65]. Our previous studies showed that PGPR inoculation increased pulegone, menthone, menthol, and linalool production in *M. piperita* [66] and in other aromatic plant species: *A. brasilense* inoculation increased the levels of ocimenone and tagetone by 71% and 66%, respectively, in *Tagetes minuta* [38]; in *Origanum majorana*, the main compounds, terpinen-4-ol, cis-sabinene hydrate, trans-sabinene hydrate, and a-terpineol were also increased by inoculation with *P. fluorescens* [67]; and greater amounts of terpineol and eugenol were reported for sweet basil inoculated plants [36].

In a previous study, we reported that *M. piperita* exposed to SA and MeJA increased the total EO yield. Particularly, external application of 1 and 2 mM SA increased the EO yield approximately two-fold, while the combination with inoculation led to a 3-fold increase compared to control plants [40]. Curiously, the main monoterpene in plants exposed to SA increased in greater proportions; particularly, menthol rose approximately 7-fold, and pulegone, linalool, limonene and menthone 3-fold in comparison with control plants. Moreover, in plants exposed to combined treatments (PGPR + SA), the levels of the main monoterpene were similar to those of exposure to SA alone. The genes involved in isoprenoid biosynthesis have been shown to be transcriptionally upregulated by SA in *Salvia miltiorhiza* and *Michelia chapensis* [68,69]. The expression of three prenyltransferases from the core terpenoid biosynthetic pathway has been shown to be upregulated by SA in different species as well [70,71,72]. Similarly, the levels of many isoprenoids, have been shown to be upregulated during drought or salt stress in parallel to increases in SA levels (see, for instance, [73,74]). In addition, holm oaks fumigated with SA showed higher monoterpene levels in leaves and enhanced volatile monoterpene emission [75]. Zhang et al. [76] reported that treatment of *Glycine max* with 1 mM SA induced transcription of a newly identified gene encoding a monoterpene synthase. Treatment of *Cistus creticus* subsp. Creticus with 5 mM SA increased the expression of two genes which encode for enzymes that catalyze the first reactions of methyl-erythritol-phosphate and mevalonate [77]. Xu et al. [78] reported that 8 days after exposure to SA, the medicinal herb, *Houttuynia cordata*, increased the accumulation of α-thujene, α-pinene, α-terpinene, β-pinene, β-myrcene, limonene, and β-ocimene. Although the complete mechanism of SA-mediated plant defense is still not completely understood, the central role of SA in plant defense is universally accepted [79]. Further research is needed to determine the effects of SA on the biosynthesis of particular terpenoids (especially in the committed steps leading to these particular terpenoids), and to establish the possible relationship between the biosynthetic pathways leading to different individual terpenoids.

In relation to the effects of MeJA on the total EO yield of peppermint, it was previously reported that it produced an increase depending on the concentration applied: 3-fold for 1 and 2 mM and 5-fold for 4 mM. The strongest effect was observed when plants were treated with 2 mM and inoculated with rhizobacteria, increasing approximately 8-fold in comparison with controls or inoculated plants, indicating that there is a synergism between PGPR and MeJA [40]. Regarding the amount of the main monoterpene evaluated, not all responded in the same way. Pulegone and menthol showed the same trend as total EOs reported, while limonene, linalool, and menthone did not differ significantly from control plants for exposure to 1 and 2 mM and approximately 4 times for 4 mM. In relation to the combination of treatments, in the present study, we observed a 10-fold increase in the amount of pulegone and menthone when combining 2 mM with inoculation with WCS417. This increase was 2-fold greater as shown in the total EO yield reported [40]. Wang and Wu [80] stated that MeJA is an effective inducer of the terpenoid in *Taxus* spp. and also induced rapid activation of PAL.

JA and its derivatives and precursors are involved in the induction of plant defense responses [81,82,83,84,85]. These hormones play a central role in the regulation of the biosynthesis of several secondary metabolites, such as alkaloids, terpenoids, phytoalexins, coumarins, anthocyanins, among others, by gene regulation, promoting an increase in the number of transcripts of the enzymes linked to the metabolic pathway of those compounds [86,87,88]. Similarly to the results obtained in the present study, Schmidt et al. [89] reported that the exogenous application of JA induced accumulation of monoterpenes and diterpenes in *Picea abies* stems. However, in this study, treatment with JA did not produce a significant increase in production of terpenoids in eucalyptus leaves, which contained abundant secretory cavities [90]. Likewise, both the emission of linalool and the content of this monoterpene in glandular trichomes of tomato plants increased after the application of JA 1 mM. Treatment with JA also increased the transcription levels of the *LeMTS1* gene, which codes for a linalool synthase located in trichomes of tomato plants [91].

Few studies have been reported on the effects of exogenous application of JAs on the production of secondary metabolites in aromatic and medicinal plants. Złotek et al. [92] reported that treatment with JA significantly increased the content of monoterpenes linalool, eugenol, and limonene in *Ocimum basilicum*. The results obtained in this work indicate that the biosynthesis of terpenoids in aromatic plants and other plant species can be induced by treatment with JA or its methylated derivative, MeJA.

The increase observed in monoterpene accumulation is correlated with an increase in the density of glandular trichomes [93,94]. Biochemical studies with isolated peltate glandular trichomes of peppermint have revealed that the secretory cells are responsible for the secretion of monoterpenes in the oil-storage space [95,96]. A previous study showed that exposure to SA and MeJA produced a 2-fold increase in the density of peltate trichomes in peppermint [40]; this explains the increase in content of the main monoterpenes observed in the present study. Moreover, peltate trichomes also serve as the site of monoterpene biosynthesis [96]. Thus, upregulation of limonene synthase *Ls* and *Pr* gene expression was observed. The biosynthesis of monoterpenes from primary metabolism requires a series of enzymatic steps. First, using geranyl diphosphate as substrate, *Ls* generates limonene and minor amounts of myrcene, alpha-pinene, and beta-pinene [96]. In the present study, an upregulation of *Ls* was observed that correlates with the increase observed in limonene content in plants exposed to 2 and 4 mM MeJA alone and 2 mM MeJA + PGPR. In contrast, no effect on *Ls* expression was observed in plants treated with SA, despite the 5-fold increase in limonene observed in those plants. A similar response was observed for pulegone reductase *Pr* gene expression; menthone and isomenthone (in a 2:1 to 10:1 ratio) were formed by the action of the NADPH-dependent *Pr* using pulegone as substrate [96]. The enzyme responsible for the synthesis of menthone was upregulated only in plants exposed to MeJA (4 mM) and in the combined treatments 2 mM MeJA + PGPR, while SA treatment did not affect *Pr* expression, despite the accumulation of menthone in SA-treated plants being increased in relation to control plants. The poor or the total lack of correlation between gene expression of *Pr* and *Ls* with the respective monoterpene content was probably because at the time that the qPCR were performed (7 days after phytohormone application) the increase in the biosynthesis of monoterpene may have already occurred, probably 48–72 h after phytormone application [97], and it is well known that the monoterpenes are stored in the glandular trichomes. On the other hand, protein stability may be increased due to post-translational modifications such as phosphorylation, acetylatylation, glycosylation, and also it could be that protein may be long lived and accumulates over time whereas mRNA turnover is quick [98].

In a previous study, we observed a significant increase in free jasmonic acid and the active form JA-Ile as well as SA in *M. piperita* plants inoculated with different rhizobacteria strains. The induction of SA and JA by rhizobacteria in the host plants suggests that plants may perceive these bacteria as a risk and thereby initiate a defensive response [40]. Elicitors such as SA and JA are considered as signal molecules that activate the signal-transduction cascade, leading to the activation and expression of genes related to the biosynthesis of secondary metabolites and playing a major role in the defense response [99]. It was reported that exogenous applications of MeJA result in the major reprogramming of genes, including genes that are known to be involved in plant stress responses [100,101], genes that induce defense by increasing activities of pathogenesis-related proteins, and genes that cause oxidative bursts, phytoalexin accumulation, lignification, and cell wall stiffening [102,103]. Furthermore, JA induces the expression of genes involved in biosynthesis, which leads to the accumulation of antimicrobial secondary metabolites, including alkaloids, terpenoids, flavonoids, anthraquinones, and glucosinolates in different plant species [104]. 

Total phenolic content has been related with stress tolerance, either through contributing with indirect photo protection or by participating directly as antioxidants [51,105]. The increase of phenolic compounds, which are considered the major antioxidant compounds in plants, resulted in a significant decrease in antioxidant capacity. Others organic compounds act as antioxidants like the monoterpenes, through the direct ROS scavenging pathway, and show a capacity for modulating the endogenous antioxidant system [106,107].

Interest in phenolic compounds has considerably increased in recent years because of their broad chemical spectrum and diverse biological properties [108]. In addition to their antioxidant properties, these compounds have been reported to be potential candidates in reducing cardiovascular diseases and anticarcinogenic activity, with antiallergenic, antiarthrogenic, antiinflammatory, antimicrobial, and antithrombotic effects [109]. Extracts of fruits, herbs, vegetables, cereals, and other plant materials rich in phenolics are increasingly of interest in the food industry, because they retard the oxidative degradation of lipids and thereby improve the quality and nutritional value of food [108].

Also, in view of the environmental, food-safety and health related issues associated with the application of chemical insecticides, growing emphasis is being laid on pest control through plant resources. In addition to the menthol and pulegone present in the essential oil of the Mentha species, which are the substances that give mints their characteristic aromas and flavors [4,110,111], they are also found to possess insecticidal, antiviral and fungicidal activities. Pulegone showed potent insecticidal activity against the mushroom scatopsid fly, *Scatopse* spp., in fumigant bioassay [112], and significant antibacterial activity is also observed for Mentha oil due to the presence of menthol [113]. These compounds form complexes with bacterial enzymes and protein and inhibit the growth of bacterial pathogens [114], causing the disruption of the plasma membrane, which increases its permeability and depolarizes its potential, finally leading to the death of the bacteria [115]. Menthol and limonene have also been reported as potential antifungal agents against plant pathogenic fungi [4].

## 4. Material and Methods

### 4.1. Plant Material, Bacterial Inoculation, and Treatments

*Pseudomonas simiae* WCS417r (formerly known as *P. fluorescens* WCS417r; [116]; *P. putida* SJ04) is a native fluorescent strain isolated from rhizospheric soil under a commercial crop of *Mentha* x *piperita* (San José) in Córdoba, Argentina, and demonstrated to have plant growth-promoting activity (GenBank KF312464.1) [117]; and *Bacillus amyloliquefaciens* GB03 (originally described as *Bacillus subtilis* GB03 [118]). 

Bacteria were grown on LB medium [119] for routine use and maintained in nutrient broth with 15% glycerol at −80 °C for long-term storage.

Each bacterial culture was grown overnight at 30 °C with rotation at 120 rpm until reaching the exponential phase, washed twice in 0.9% NaCl with centrifugation (4300× *g*, 10 min, 4 °C), resuspended in sterile water, and adjusted to a final concentration of ~10^8^ CFU/ mL for use as inoculum. 

Plants were grown in plastic pots (diameter 12 cm, depth 22 cm) containing sterilized vermiculite. *M.* x *piperita* seedlings were planted (one per pot) in vermiculite and inoculated with 1000 µL bacterial suspension. Four experimental treatments were performed with the bacteria: sterile water (control), SJ04, WCS417r, and GB03. 

### 4.2. Greenhouse Experiments

*M.* x *piperita* in vitro micropropagation was performed as described by Cappellari et al. [66]. On day 7 of culture, obtained by in vitro multiplication, they were transplanted directly into vermiculite in a greenhouse, and watered by a micro-irrigation system. All plants received Hoagland’s nutrient medium (20 mL/pot) twice per week [38]. Plants were grown in a growth chamber under controlled conditions of light (16/8-h light/dark cycle), temperature (22 ± 2 °C), and relative humidity (~70%). Bacterial suspensions as described above were applied to experimental seedlings, and sterile water was applied to control seedlings. After 7 days of inoculation, plants were sprayed until run-off with 1, 2, or 4 mM methyl jasmonate solution (MeJA) (Sigma–Aldrich, St. Louis, MO, USA, 1% methanol in water, *v*/*v*) or 1 or 2 mM SA solution (1% ethanol in water, *v*/*v*). The plants were left to dry for 30–60 min. For the phytohormone control treatments, a solution of the solvent used was applied. After phytohormone or control treatments, plants were transferred to a climate chamber with the phytohormone treatments spatially separated from other treatments because MeJA is very volatile.

After 14 days of applied phytohormone treatments, plants were removed from pots. Experiments were replicated 3 times (10 pots per treatment; 1 plant per pot) and were performed under non-sterile conditions.

### 4.3. Determination of Total Phenolic Content

Total phenols were determined using Folin–Ciocalteu reagent [120]. Each plant extract (0.5 mL) or gallic acid (standard phenolic compound) was mixed with Folin–Ciocalteu reagent (0.5 mL, diluted with 8 mL distilled water) and aqueous Na_2_CO_3_ (1 mL, 1 M). After 1 h, the level of total phenols was determined by colorimetry at a wavelength of 760 nm. Total phenol values were expressed in terms of µg gallic acid (a common reference compound) equivalent per g plant dry weight [38].

### 4.4. Determination of PAL Enzyme Activity

100 mg mint leaves were homogenized with liquid nitrogen using a mortar and pestle containing appropriate buffer solution (50 mM potassium phosphate and 1 mM EDTA, pH 7.8) and 1% PVP (polyvinylpyrrolidone) and then filtered through a 0.20 mm nylon filter into a centrifuge tube. The tissue extract was centrifuged at 12,000× *g* for 40 min at 4 °C. The supernatant to be used for enzyme activity determination was stored at 20 °C. Protein concentration was determined by the method described by Bradford [121].

PAL activity was assayed following the method described by Beaudoin–Eagan and Thorpe [122] by measuring the amount of trans-cinnamic acid formed at 290 nm. The reaction mixture consisted of 100 µL of enzyme extract, 900 µL 6 mM of l-phenylalanine, and 500 mM Tris HCl buffer solution (pH 8). The mixture was placed in a water bath at 37 °C for 70 min, and the reaction was stopped by the addition of 50 µL of 5 N HCl. Trans-cinnamic acid (1 mg/mL was used as standard and PAL activity was expressed as μg trans-cinnamic acid/min mg protein.

### 4.5. Extraction and Quantification of Main Monoterpene EO Components

Shoot samples were individually weighed and subjected to hydrodistillation in a Clevenger-like apparatus for 40 min. The volatile fraction was collected in dichloromethane, and β-pinene (1 μL in 50 μL ethanol) was added as an internal standard.

The major *M. piperita* EO components, which make up ~60% of total oil volume, are limonene, linalool, (−)-menthone, (−)-menthol, and (+)-pulegone. These compounds were quantified with respect to the standard added during the distillation procedure. The oil components were initially identified based on mass spectral and retention time data and confirmed by direct comparisons with commercial standards from Sigma–Aldrich Co. Flame ionization detector (FID) response factors for each compound generated essentially equivalent areas (differences <5%). Chemical analyses were performed as reported by Banchio et al. [36].

### 4.6. Total RNA Extraction and Quantitative Real-Time PCR

Total RNA from 50 mg lyophilized plant material was isolated using the Plant RNA Isolation Kit (Stratec, Berlin, BE, Germany), according to the manufacturer’s instructions but including an additional DNA digestion step (RNase Free DNase set (Qiagen, Valencia, CA, USA). Using identical amounts of total RNA, template cDNA for subsequent PCR reactions was generated using Superscript™ III (Invitrogen, Karlsruhe, BW, Germany) according to the manufacturer’s instructions. Quantitative real-time PCR was performed with SsoAdvanced Universal SYBR Green Supermix (BIO-RAD, Munich, Bavaria, Germany) and 10 pmol forward and 10 pmol reverse primer. 

Relative RNA levels were calibrated and normalized with the level of housekeeping gene actin. Primer sequences for *Actin* (Act), *Limonene synthase* (Lim S), and *Pulegone reductase* (Pr) are shown in Table 3. PCR was performed using a CFX Connect Real-Time PCR system (BIO-RAD) according to the instruction manual. Transcript abundance was normalized to the transcript abundance of the actin.

### 4.7. Statistical Analyses

Data were pooled and subjected to analysis of variance (ANOVA) followed by comparison of multiple treatment levels with controls using the Tukey test and principal component analysis (PCA). Control with solvent for MeJA and SA did not differ statistically with the control and is therefore not shown in the figures. For Figure 1, Figure 2, Figure 3 and Figure 4, the statistics were performed on the native data, but the figures were made using “fold changes” in order to facilitate the interpretation of the results. Differences between means were considered significant for *p* values <0.05. The Infostat software program, v. 2008 (Group Infostat, Universidad Nacional de Córdoba, Córdoba, Argentina), was used for all statistical analyses. In the Supplementary Tables S1 and S2, the native data is shown.

## 5. Conclusions

Due to the multiple properties of secondary metabolites from *M. piperita*, monoterpene and phenolic compounds arouse the interest of the pharmaceutical and food industry as well as cosmetics producers. Elicitation of secondary metabolites appears to be a promising and innovative alternative; there are combinations of both biotic (PGPR) and abiotic (phytohormone) forms of elicitors that stimulate metabolism, and there have been many studies of their potential use individually for improving secondary metabolism biosynthesis, but no reports were found with the combination of both. This study revealed that peppermint plants treated with elicitors SA or JA and simultaneously inoculated with PGPR could enhance the production of phenolic compounds and monoterpenes. Considering the different concentrations of SA and MeJA evaluated, we suggest using a concentration of MeJA 2 mM for the external application on *M. piperita* 7 days before harvest. This is a cost-effective concentration, which increased the main secondary metabolite content, and taking into account the fact that a concentration of MeJA 4 mM is more expensive, not necessarily any more effective, and did not increase the main monoterpene by as much as the 2mM concentration. This is the first report demonstrating that inoculation with PGPR in combination with an external phytohormone increases phytochemical production in relation to each treatment alone. Results from this study will help improve secondary metabolite production for this crop.

## Figures and Tables

**Table 1:** Effects of inoculation with PGPR strains and external application of SA on concentration of main shoot essential oil components of *M. × piperita* plants (mean ± SE). Means followed by the same letter in a given column are not significantly different according to the Tukey test (*p* < 0.05).

Treatments	Linalool (µg/g fw)	(−)-Limonene (µg/g fw)	(−)-Menthone (µg/g fw)
Control	0.15 ± 0.01 a	0.20 ± 0.02 a	0.50 ± 0.03 a
G	0.61 ± 0.07 b	0.76 ± 0.06 b	1.15± 0.05 bc
S	0.48 ± 0.08 b	0.73 ± 0.08 b	1.34 ± 0.15 c
W	0.52 ± 0.05 b	0.65 ± 0.07 b	0.99 ± 0.09 b
Control for SA	0.14 ± 0.02 a	0.19 ± 0.04 a	0.43 ± 0.09 a
1 mM SA	0.54 ± 0.07 b	1.09 ± 0.18 b	1.30 ± 0.09 b
2 mM SA	0.40 ± 0.07 ab	1.01 ± 0.20 b	1.40 ± 0.09 b
1 mM SA + G	0.41 ± 0.03 cd	0.91 ± 0.06 b	1.34 ± 0.07 b
1 mM SA + S	0.32 ± 0.03 ab	0.99 ± 0.18 b	1.71 ± 0.35 c
1 mM SA + W	0.40 ± 0.05 ab	0.66 ± 0.17 ab	1.45 ± 0.05 b
2 mM SA + G	0.34 ± 0.09 ab	1.14 ± 0.03 b	1.50 ± 0.22 b
2 mM SA + S	0.41 ± 0.05 ab	1.18 ± 0.08 b	1.53 ± 0.06 b
2 mM SA + W	0.22 ± 0.06 a	1.06 ± 0.17 b	1.31 ± 0.05 b

G—GB03; S—SJ04; W— WCS417; SA—Salicylic Acid.

**Table 2:** Effects of direct inoculation with PGPR strains and external application of MeJA on concentration of main shoot essential oil components of *M. x piperita* plants (mean ± SE). Means followed by the same letter in a given column are not significantly different according to Fisher’s LSD test (*p* < 0.05).

Treatments	Linalool (µg/g fw)	(−)-Limonene (µg/g fw)	(−)-Menthone (µg/g fw)
Control MeJA	0.15 ± 0.02 a	0.25 ± 0.02 a	0.35 ± 0.03 a
1 mM + MeJA	0.16 ± 0.02 a	0.25 ± 0.05 a	0.43 ± 0.08 a
2 mM + MeJA	0.22 ± 0.04 a	0.21 ± 0.04 a	0.47 ± 0.13 a
4 mM + MeJA	0.97 ± 0.12 c	0.83 ± 0.07 b	1.42 ± 0.14 b
1 mM MeJA + G	0.23 ± 0.04 a	0.40 ± 0.05 a	0.23 ± 0.03 a
1 mM MeJA + S	0.32 ± 0.07 a	0.25 ± 0.03 a	0.51 ± 0.13 a
1 mM MeJA + W	0.35 ± 0.07 ab	0.37 ± 0.04 a	0.44 ± 0.13 a
2 mM MeJA + G	0.79 ± 0.15 bc	1.09 ± 0.07 bc	1.75 ± 0.17 bc
2 mM MeJA + S	1.12 ± 0.17 c	1.23 ± 0.07 c	1.95 ± 0.17 bc
2 mM MeJA + W	0.84 ± 0.21 c	1.11 ± 0.10 bc	2.54 ± 0.45 c
4 mM MeJA + G	0.21 ± 0.08 a	0.22 ± 0.04 a	0.27 ± 0.07 a
4 mM MeJA + S	0.20 ± 0.02 a	0.23 ± 0.01a	0.20 ± 0.04 a
4 mM MeJA + W	0.23 ± 0.02 a	0.27 ± 0.09 a	0.42 ± 0.12 a

G—GB03; S—SJ04; W—WCS417; MeJA—Methyl Jasmonate.

**Table 3:** Primer sequences for RT-PCR.

Gene	Forward Primer Sequence (5′-3′)	Reverse Primer Sequence (5′-3′)
*Act*	GCTCCAAGGGCTGTGTTCC	TCTTTCTGTCCCATGCCAAC
*Ls*	TTGTGGCGAATTCTCTCGCT	GGCTTCTGAGCTGGTCACTT
*Pr*	GCATGGAGATCCCAGATGGC	AGTAGAGCCAGGAAGGATGGA

## Supplementary Materials

Supplementary materials can be found at https://www.mdpi.com/1422-0067/21/1/50/s1.

## Abbreviations

EO	essential oil
PGPR	plant growth-promoting rhizobacteria
JA	jasmonic acid
MeJA	methyl jasmonate
SA	salicylic acid
TPC	total phenolic content
PAL	phenylalanine ammonia lyase
