# Supplementary material for: Improving Phenolic Total Content and Monoterpene in Mentha x piperita by Using Salicylic Acid or Methyl Jasmonate Combined with Rhizobacteria Inoculation
Source: Int J Mol Sci. 2019 Dec 19;21(1):50. doi: 10.3390/ijms21010050 (PMC6981552; doi:10.3390/ijms21010050)
Supplement: Supplementary file 1 [file ijms-21-00050-s001.pdf]

International Journal of *Molecular Sciences*

MDPI

*Supplementary*

# Improving phenolic total content and monoterpene in *Mentha* x *piperita* by using salicylic acid or methyl jasmonate combined with rhizobacteria inoculation

**Lorena del Rosario Cappellari [1], Maricel Valeria Santoro [2], Axel Schmidt [2], Jonathan Gershenzon [2] and Erika Banchio [1,*]**

[1] INBIAS (CONICET-Universidad Nacional de Río Cuarto), Campus Universitario, 5800 Río Cuarto, Argentina

[2] Department of Biochemistry, Max Planck Institute for Chemical Ecology, Hans-Knöll-Str. 8, 07745 Jena, Germany

* Correspondence: ebanchio@exa.unrc.edu.ar; Fax: 0054-358-4676232

**Supplementary Table S1.** Effects of inoculation with PGPR strains and of the external application of SA on TPC, PAL activity, pulegone and menthol content of *M.* x *piperita* plants. Values are mean ± standard error (SE).

| Treatments | TPC (µg/g fw) | PAL activity (µg Trans-Cinnamic Acid/Min × mg Protein) | Pulegone (µg/g fw) | Menthol (µg/g fw) |
|---|---|---|---|---|
| Control | 209.84 ± 13.20 | 4.62 ± 0.30 | 3.74 ± 0.16 | 0.20 ± 0.02 |
| G | 333.51 ± 32.84 | 13.84 ± 2.21 | 13.90 ± 0.71 | 1.29 ± 0.10 |
| S | 329.62 ± 27.73 | 16.84 ± 2.38 | 10.75 ± 0.83 | 1.22 ± 0.10 |
| W | 347.98 ± 22.38 | 11.30 ± 2.30 | 9.71 ± 0.42 | 0.99 ± 0.28 |
| Control SA | 254.03 ± 24.95 | 4.63 ± 0.30 | 4.27 ± 0.27 | 0.19 ± 0.02 |
| 1 mM SA | 451.60 ± 29.96 | 51.79 ± 5.89 | 10.78 ± 1.44 | 1.36 ± 0.13 |
| 2 mM SA | 443.06 ± 48.55 | 58.38 ± 3.92 | 8.12 ± 1.23 | 1.48 ± 0.21 |
| 1 mM G | 458.13 ± 15.21 | 61.49 ± 10.61 | 11.11 ± 1.82 | 1.63 ± 0.15 |
| 1 mM S | 419.43 ± 8.98 | 58.13 ± 9.22 | 12.05 ± 1.51 | 1.40 ± 0.20 |
| 1 mM W | 446.32 ± 14.42 | 81.63 ± 13.16 | 10.90 ± 1.56 | 1.54 ± 0.26 |
| 2 mM G | 672.22 ± 7.84 | 121.36 ± 15.99 | 6.56 ± 0.82 | 1.81 ± 0.10 |
| 2 mM S | 461.65 ± 39.77 | 99.88 ± 10.54 | 8.63 ± 1.52 | 1.70 ± 0.55 |
| 2 mM W | 397.95 ± 16.33 | 69.96 ± 26.38 | 9.36 ± 1.45 | 1.75 ± 0.41 |

**Supplementary Table S2.** Effects of inoculation with PGPR strains and of the external application of MeJA on TPC, PAL activity, pulegone and menthol content of *M.* x *piperita* plants. Values are mean ± standard error (SE).

| **Treatments** | ***TPC* (μg/g *fw*)** | ***PAL activity* (*μg trans-cinnamic acid min-1 mg-1 protein*)** | ***Pulegone* (μg/g fw)** | ***Menthol* (μg/g fw)** |
|---|---|---|---|---|
| Control MeJA | 192.43 ± 16.52 | 4.60 ± 0.28 | 3.99 ± 0.28 | 0.23 ± 0.07 |
| 1mM | 320.21 ± 13.05 | 90.54 ± 10.07 | 10.53 ± 0.99 | 0.75 ± 0.18 |
| 2mM | 440.41 ± 22.72 | 142.70 ± 25.97 | 8.94 ± 1.11 | 0.69 ± 0.15 |
| 4mM | 473.42 ± 53.64 | 280.98 ± 25.22 | 16.61 ± 1.91 | 1.56 ± 0.14 |
| 1mM + G | 321.30 ± 11.24 | 139.44 ± 14.54 | 5.85 ± 0.40 | 0.42 ± 0.06 |
| 1mM + S | 304.73 ± 12.06 | 134.93 ± 5.75 | 9.67 ± 0.53 | 0.47 ± 0.17 |
| 1mM + W | 316.45 ± 24.25 | 115.45 ± 22.93 | 9.27 ± 0.89 | 0.78 ± 0.11 |
| 2mM + G | 401.43 ± 18.72 | 192.39 ± 13.98 | 22.55 ± 2.06 | 1.76 ± 0.32 |
| 2mM + S | 379.10 ± 16.73 | 176.66 ± 37.48 | 30.12 ± 1.32 | 2.31 ± 0.37 |
| 2mM + W | 392.65 ± 49.40 | 165.60 ± 40.41 | 35.89 ± 1.18 | 2.36 ± 0.47 |
| 4mM + G | 613.11 ± 25.30 | 301.73 ± 61.09 | 9.71 ± 0.82 | 0.61 ± 0.13 |
| 4mM + S | 563.22 ± 17.49 | 282.95 ± 39.37 | 8.06 ± 0.43 | 0.58 ± 0.09 |
| 4mM + W | 481.25 ± 21.30 | 276.18 ± 25.45 | 10.30 ± 0.88 | 0.76 ± 0.02 |
